# Effects of visual standard channel combined with visual superfine precision puncture channel or super-mini channel percutaneous nephrolithotomy on multiple renal calculi

**DOI:** 10.12669/pjms.343.14567

**Published:** 2018

**Authors:** Wenzeng Yang, Zhenyu Cui, Tao Ma, Chunlin Zhao, Hongyue Zhou, Jingyang Guo

**Affiliations:** 1 Wenzeng Yang, Department of Urinary Surgery, Affiliated Hospital of Hebei University, Baoding 071000, P. R. China; 2 Zhenyu Cui, Department of Urinary Surgery, Affiliated Hospital of Hebei University, Baoding 071000, P. R. China; 3 Tao Ma, Department of Urinary Surgery, Affiliated Hospital of Hebei University, Baoding 071000, P. R. China; 4 Chunlin Zhao, Department of Urinary Surgery, Affiliated Hospital of Hebei University, Baoding 071000, P. R. China; 5 Hongyue Zhou, Department of Urinary Surgery, Affiliated Hospital of Hebei University, Baoding 071000, P. R. China; 6 Jingyang Guo, Department of Urinary Surgery, Affiliated Hospital of Hebei University, Baoding 071000, P. R. China

**Keywords:** Visual standard channel, Superfine precision puncture, Super-mini channel, Multiple renal calculi

## Abstract

**Objective:**

To compare the therapeutic effects of visual standard channel combined with visual superfine precision puncture channel or super-mini percutaneous nephrolithotomy (PCNL) on multiple renal calculi.

**Methods:**

A total of 86 patients with multiple renal calculi were retrospectively analyzed. According to different working channels, they were divided into a visual puncture channel group (visual puncture standard channel combined with visual superfine precision puncture channel, n=38) and a conventional puncture channel group (standard channel combined with super-mini channel, n=48). The two groups were compared in terms of time of channel establishment, surgical time, reduction of hemoglobin, phase I clearance rate of calculi, and surgical complications.

**Results:**

The time of establishing visual/conventional standard channel was (4.5±1.5) min vs. (6.8±1.8) minutes (t=6.326, P=0.000), and the time of establishing visible superfine/super-mini channel was (4.52±0.97) minutes vs. (7.76±1.35) minutes (t=2.017, P=0.000). The surgical time was (92±15) minutes vs. (115±13) minutes (t=26.640, P=0.000). The Phase-I clearance rate was 86.7% (33/38) vs. 87.5% (42/48) (χ^2^=0.008, P=0.928), the reduction of hemoglobin was (12.21±2.5) g/L vs. (13.22±3.5) g/L (t=2.017, P=0.137), the blood transfusion rate was 13.16 (5/38) vs. 8.33% (4/48) (χ^2^=0.006, P=0.941), the postoperative fever rate was 7.89% (3/38) vs. 14.58 (7/48) (χ^2^=0.006, P=0.941), and the hospitalization stay length was (6.5±1.0) vs. (6.6±1.2) (t=0.413, P=0.681). There were no significant differences between the two groups.

**Conclusion:**

Both surgical approaches had high clearance rates of multiple renal calculi, safety, reliability and few complications. However, compared with the conventional puncture channel, the visual one was easy to operate and dramatically shortened the time of establishment, thus being safer and more accurate.

## INTRODUCTION

Standard channel percutaneous nephrolithotomy (PCNL) has been widely used in the treatment of calculus of upper urinary tract and has become a preferred method for complex nephrolithiasis including staghorn renal calculi and multiple renal calculi.^1^ The PCNL combined with minimally invasive percutaneous nephrolithotomy (MPCNL) in the treatment of complicated calculus of upper urinary tract can significantly improve the lithotripsy removal efficiency.^2^ On this basis, we used the visual puncture standard channel (F24) EMS ultrasonic lithotripsy combined with visual superfine channel (F4.8) target puncture holmium laser lithotripsy and standard channel (F24) combined with super-mini channel (F18) PCNL EMS ultrasonic lithotripsy in the treatment of multiple renal calculi, to compare the effects and complications of the two surgeries.

## METHODS

### Baseline clinical data

This study has been approved by the ethics committee of our hospital, and written consent has been obtained from all patients. A total of 86 patients were selected. There were 38 cases in the visual puncture channel group, including 20 males and 18 females, with a mean age of 38.9 (25~70) years old and the size of calculi of 15 mm × 40 mm (12 mm × 25 mm to 20 mm × 45 mm) on average. There were 48 cases in the conventional puncture channel group, including 29 males and 19 females, with a mean age of 40.6 (23~72) years old and the size of calculi of 18 mm × 38 mm (15 mm × 25 mm to 25 mm × 50 mm). Then 15 cases of staghorn calculi, 61 cases of multiple renal calculi (9 cases on bilateral sides) and 10 cases of unilateral renal calculi were confirmed through the urinary system color ultrasound, KUB, CTU and IVU examinations. Before this study, 10 patients had received PCNL surgery, and seven had received ESWL. The two groups had similar baseline clinical data (P>0.05) (Table-I).

**Table-I:** Baseline clinical data (*x̄*±s)

Group	Age	Gender		Staghorn calculi		Multiple calculi in kidney calices	Maximum diameter of calculi (mm)	History of calculi removal	
	
	(year)	Male	Female	Complete	Partial			ESWL	PCNL
Visual puncture channel (n=38)	38±13	20	18	3	4	21	25±16	3	4
Conventional puncture channel (n=48)	40±15	29	19	3	5	40	28±14	4	6
t (χ^2^)	*t*=0.651	χ^2^=2.128		χ^2^=0.000			*t*=0.926	χ^2^=0.014	
P	0.517	0.145		1.000			0.357	0.906	

### Inclusion criteria

With PCNL surgical indications; age ≥18 years old. Exclusion criteria: Uncontrollable infective calculi, pyonephrosis; congenital abnormalities in kidneys; uncontrolled systemic bleeding disorders; wandering and grafted kidneys, and severe renal ptosis; uncontrollable diabetes or severe cardiopulmonary dysfunction, unable to tolerate surgery.

### Surgical methods

After the epidural anesthesia, the patient was placed in a lithotomy position at first; a zebra urological guide-wire (Germany LAKH ST-32150) was placed in the operated ureter under the ureteroscope, the guide-wire was retained, then the ureteroscope was taken out; an F5 ureteral catheter was inserted into the ureter inversely along the guide-wire, and then the guide-wire was pulled out. An F16 Foley catheter was placed in the bladder, with both external parts fixed to prevent them from falling off. The far end of the ureteral catheter was connected with pressurized 0.9% saline to establish artificial hydronephrosis. Then the position of the patient was changed into a prone position, the renal region was under-laid and fixed; the 11th costal tip, the 12th intercostals, and the position between the posterior axillary line and the linea scapularis were chosen, which was the nearest to the puncture point of the target renal calyx.

### Visual puncture channel group

A visual standard channel and a visual superfine precision puncture channel were established through the F4.8 visual puncture nephroscope system. The B ultrasonic guided system was connected with the visual puncture mini-nephroscope system which was placed in the visual puncture sheath. After measuring the optimum location and depth by ultrasound, the visual puncture needle was guided to puncture under direct vision. Until the calculi could be seen in the monitor, the puncture was successful. The puncture sheath was retained, the visual nephroscope pulled out, and the zebra urological guide-wire embedded. An F8 fascial dilator was led along the guide-wire to expand the fascia. Then the skin was cut open for about 1.5 cm to place a balloon dilatation catheter (America BARD X-FORCE). The full pressure pump system (America BARD, 20 ml) was connected, and stroke-physiological saline solution was injected by the pressure pump until the pressure was up to 25 mmHg (1 mmHg = 0.133 kPa). After expanding the balloon fully for 3min, the matched F24 puncture sheath was screwed in, the balloon dilatation catheter was removed after decompression, and then the visual percutaneous standard channel was established successfully. Then a Wolf F20.8 nephroscope was placed to smash and clear away the calculi with the third generation of EMS ultrasonic pneumatic lithotripsy system. According to the ultrasonic examination result and the distribution of renal calices, a visual percutaneous superfine channel was established for residual calculi, and the location, depth and perspective of the residual calculi were confirmed with B-mode ultrasound again; the visual puncture needle was led to puncture under direct vision, and then the mini-nephroscope was pulled out until the residual calculi were found; then the zebra urological guide-wire was led in, and the superfine channel sheath was retained along the guide-wire (F4.8). Finally, a 200μm holmium laser fiber (Lumenis holmium laser 75W) was led in to flush the catheter and smash the calculi at the same time. The calculi with a size of about 3-5mm were sucked out simultaneously through the original visual standard channel.

### Conventional puncture channel group

An 18G puncture needle was led in to puncture the target renal calyx. After pulling out the core of the needle, if there was some urine flowing out, the puncture was successful. Then a zebra urological guide-wire was embedded to record the depth of the puncture needle, and an F8 fascial dilator was inserted along the guide-wire. Then the skin was cut open for about 1.5 cm with a sharp knife, a balloon dilatation catheter (America BARD X-FORCE) was embedded along the zebra urological guide-wire, the far end was connected with the full pressure pump system (America BARD, 20 ml) to inject the stroke-physiological saline solution by the pressure pump until the pressure was up to 25 mmHg (1 mmHg = 0.133 kPa). After expanding the balloon fully for three minutes, the matched F24 puncture sheath was screwed in, the balloon dilatation catheter was removed after decompression, and then the visual percutaneous standard channel was established successfully. Then a Wolf F20.8 nephroscope was placed to smash and clear away the calculi with the third generation of EMS ultrasonic pneumatic lithotripsy system. According to the ultrasonic examination result and the distribution of renal calices, a super-mini channel was established for the residual calculi of the target renal calyx, an F18 channel sheath was retained for the broken calculi, and then the calculi were smashed and cleared away with the third generation of EMS ultrasonic pneumatic lithotripsy system. The catheter was flushed and the calculi were smashed at the same time. The size of calculi was about 3-5 mm, and the bigger ones were sucked out through the original standard channel.

After surgery, an F5 double J tube and an F18 nephrostomy tube was retained in both groups through the standard channel, the superfine channel/super-mini channel was applied with band-aids, and the drainage tube was not retained. The catheter was retained for 1 to 2 days, and a review was performed by KUB or CT 1 to 2 days after the surgery to learn about the smashing and discharge of calculi and the position of the double J tube. If the size of fragments was >4 mm, they could be powered by holmium laser through the visual superfine channel in the phase II treatment one week later. The nephrostomy tube was pulled out 5 to 7 days after the surgery, and the double J tube was pulled out four to six weeks later.

Evaluation of therapeutic effects: The patients were reviewed by KUB or CT within 4 to 6 weeks after the surgery. If there was no residual stone or the size of residual fragments was <4 mm without clinical symptoms, the calculi were deemed to be cleared away successfully.

### Observation indices

The indices included the time of channel establishment (from the beginning of B ultrasound positioning to the completion of channel establishment), surgical time (from the beginning of puncture to the completion of placing nephrostomy tube after lithotripsy), phase-I clearance rate of calculi, reduction of hemoglobin, blood transfusion rate, fever rate and hospitalization stay length.

### Statistical analysis

All data were analyzed by SPSS19.0. The categorical data were expressed as X̄±s. The categorical data conforming to normal distribution were subjected to the independent samples t-test. Rates between groups were compared by the Chi-square test. P<0.05 was considered statistically significant.

## RESULTS

The two groups had significantly different times of channel establishment and surgical times (P<0.05). Nevertheless, their phase-I clearance rates of calculi, reductions of hemoglobin, blood transfusion rates, postoperative fever rates (<38°C) and hospitalization stay lengths were similar (P>0.05) (Table-II). All patients were free from complications such as delayed bleeding, hyperpyrexia, pleural effusion and ureteral injury (Table-III). They were followed up for nine months on average (3~12), without complications such as relapsed urinary tract infection, renal insufficiency and ureteral stenosis.

**Table-II:** Surgical outcomes (*x̄*±s)

Group	Establishment time (min)		Surgical time (min)	Phase-I clearance rate
	Standard channel	Superfine/super-mini channel		
Visual puncture channel (n=38)	4.5±1.5	4.52±0.97	92±15	86.7% (33/38)
Conventional puncture channel (n=48)	6.8±1.8	7.76±1.35	115±13	87.5% (42/48)
t (χ^2^)	*t*=6.326	*t*=2.017	*t*=26.640	χ^2^=0.008
P	0.000	0.000	0.000	0.928

**Table-III:** Surgical complications (*x̄*±s)

Group	Reduction of hemoglobin (g/L)	Blood transfusion rate (%)	Postoperative fever rate (%)	Hospitalization stay length (d)
Visual puncture channel (n=38)	12.21±2.5	7.89 (3/38)	13.16 (5/38)	6.5±1.0
Conventional puncture channel (n=48)	13.22±3.5	8.33 (4/48)	14.58 (7/48)	6.6±1.2
t (χ^2^)	*t*=2.017	χ^2^=0.006	χ^2^=0.006	*t*=0.413
P	0.137	0.941	0.941	0.681

## DISCUSSION

Complicated calculi are common clinically, which is easy to be complicated by renal insufficiency, urinary tract infection and relapse, for which the stone clearance rate of any single channel is low.^3^ Standard channel PCNL has become the preferred minimally invasive surgical approach. In recent years, with the development of minimally invasive techniques, PCNL, MPCNL, super-mini PCNL (SMP)) or multi-lens combined treatment are often used for the treatment of complicated renal calculi, with a success rate of 64.3~89%.^4^-^8^ For staghorn calculi with a diameter ≥3 cm, standard channel PCNL should be first considered. The best indication of MPCNL is renal calculi and pediatric renal calculi with a diameter of ≤2 cm.^9^

To reduce the risk of channel puncture and to shorten the puncturing time, we visualized the puncture channel during PCNL. And we applied the ultrasound guide F4.8 visual puncture nephroscope system in the treatment of renal calculi. This system can realize the real-time recording of the position of the needle tip through the ultrasonic probe with the sensor and the puncture needle. After positioning the target calyceal calculus, the needle tip label and the puncture trajectory of puncture needle in the tissue can be seen on the screen of the ultrasonic display during puncture, and the perspective, depth and location of the tip can be always observed. The front of the superfine nephroscope sheath is a transparent sheath, so it can be observed on the monitor that the nephroscope passes through fat tissue, muscle tissue, perirenal fat and kidney until the calculi are found, while avoiding blood vessels or penetrating the collection system. Comparing the time of establishing the puncture channel from the two groups, the puncturing time of the visible puncture channel group was significantly shorter than that of the conventional puncture group. However, the total surgery time of the former group was longer than that of the latter group. It can be related to the reason that superfine channel (F4.8) can only be applied with holmium laser lithotripsy, whose efficiency is lower than that of ultrasound pneumatic lithotripsy which is used in super-mini channel (F18) lithotripsy. During the superfine channel holmium laser lithotripsy, the standard channel was still retained in the kidney, which could act as drainage. Holmium laser could smash the calculi into a size of 3-5 mm, which were sucked out through the standard channel, with a higher efficiency compared with powdered lithotripsy.

The phase-I multi-lens and multi-channel combined approach via ultrasound navigation guide visual channel for the treatment of multiple renal calculi can avoid weaknesses and shorten the time of channel establishment. In the process of combined lithotripsy, the standard access is smooth and the renal pelvis is maintained in a low-pressure state during the flushing of calculi in super-mini and superfine channel. Meanwhile, renal pelvis can also maintain in a low-pressure state during the negative-pressure ultrasonic lithotripsy, which can effectively prevent retrograde infection of bacteriuria, reduce postoperative fever, bacteremia and other complications. The sheath of visual puncture is only 4.8F to the maximum, almost no damage to the kidneys. There was no significant difference between the two groups in stone clearance rate, reduced hemoglobin value, blood transfusion rate and hospital stays.

In summary, the visual standard channel combined with visual superfine precision puncture channel and standard channel combined with super-mini channel PCNL for the treatment of multiple renal calculi have the advantages of high rate of stone clearance, and is safety and reliable, with less complications. However, the method of establishment of visual puncture channel for the establishment of standard channel and superfine channel can significantly shorten the set-up time and be easy to operate, which makes the clinical puncture operation safer and more accurate. Furthermore, the application of this new device and technology has also greatly shortened the learning curve for beginners of PCNL, which is conducive to the promotion of minimally invasive technology in primary hospitals.

### Authors’ Contributions

**WY & ZC** designed this study and wrote this manuscript.

**WY, TM, CZ, HZ & JG** performed this study.

**TM, CZ, HZ & JG** collected and analyzed clinical data.
